# Dr. V. Shanta: A Beacon of Hope for Indian Oncology

**DOI:** 10.7759/cureus.70011

**Published:** 2024-09-23

**Authors:** Saravanan M, Jothi Feula, Anandhalakshmi S

**Affiliations:** 1 Physiology, All India Institute of Medical Sciences, Madurai, Madurai, IND

**Keywords:** cancer, dr. v. shanta, historical vignette, indian oncology, oncology

## Abstract

Dr. V. Shanta was a pioneer in the field of oncology in India. Born into a family known for scientific contributions, she was inspired to pursue medicine and eventually joined the Cancer Institute in Chennai in 1955. Her efforts transformed the institute into a world-renowned center for cancer treatment, research, and education. Dr. Shanta’s work focused on making cancer care accessible and affordable, particularly for the underprivileged, and made possible the establishment of regional cancer centers to reach rural and remote populations. She was also instrumental in introducing modern cancer therapies in India, including chemotherapy and radiation therapy, and tailored treatment protocols for Indian patients. Her work in cancer research was phenomenal and influenced national cancer policies. As a leader, she served on several expert committees, including those of the World Health Organization, and was honored with India’s highest civilian awards for her contributions to medicine. Beyond her professional achievements, Dr. Shanta was a compassionate healer who believed in holistic care. She addressed the physical, emotional, and psychological needs of her patients and left a lasting impact on patients. Dr. V. Shanta’s life and work continue to inspire generations and her legacy remains a beacon of hope in the fight against cancer.

## Introduction and background

**Figure 1:**
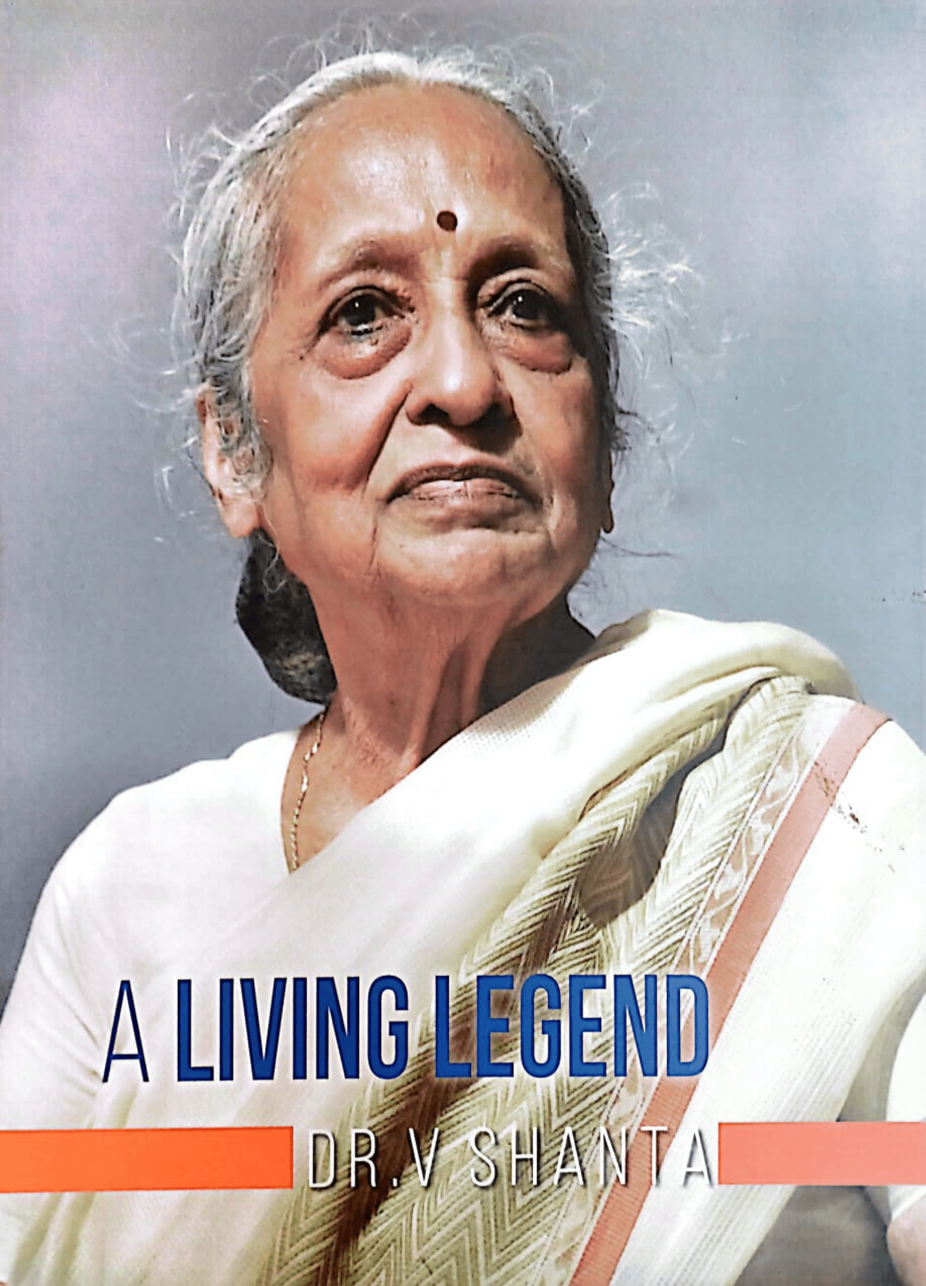
Dr. V. Shanta. Permission to use the image was obtained from the Cancer Institute (WIA).

Dr. V. Shanta, an iconic figure in Indian medicine, was a trailblazer in the field of oncology. India, a country where access to medical services had been limited for many people in those days, her life was dedicated to serving cancer patients and advancing cancer care. Through her efforts, Dr. Shanta not only transformed cancer treatment but also ensured that it became more accessible and affordable to the underprivileged. This article recollects her life and achievements and explores her legacy in Indian and global oncology (Figure 1).

## Review

Early life

Born to Viswanathan and Balaparvati on March 11, 1927, in Madras (currently Chennai), Dr. Shanta grew up in a family familiar with science and medicine. Her immediate grand uncle Sir C. V. Raman and her maternal uncle Dr. Subramaniam Chandrashekhar were Nobel Laureates [1,2]. This background shaped her passion for science and medicine from an early age. After intermediate schooling, she waited for a year to meet her age requirement to begin her educational journey at the Madras Medical College, where she obtained her MBBS degree in 1949. Her initial interest in gynecology soon shifted to oncology due to her maternal uncle’s pioneering work in cancer surgery. In 1955, she joined the Cancer Institute in Chennai, an institution that was then in its budding stages. The Cancer Institute was established by Dr. Muthulakshmi Reddy in 1954 and was the first of its kind in India [3]. When Dr. Shanta joined, the institute was still a nascent one with limited resources and infrastructure [4]. She began her lifelong commitment to the fight against cancer there. She was instrumental in building it into a premier institution for holistic care for cancer, research, and education not just in India but globally. Her early years at the Cancer Institute were characterized by long hours, dedication, and an empathetic commitment to her patients. She worked closely with Dr. Krishnamurthi and Dr. Reddy to establish a comprehensive cancer care model that integrated treatment. Eventually, the model became a blueprint for other cancer centers across the country.

Legendary work in oncology

Dr. Shanta’s contributions to oncology were vast. She was a pioneer in introducing several advanced cancer treatment modalities in India, including radiation therapy, chemotherapy, and pediatric oncology. She played a crucial role in acquiring the first cobalt-60 teletherapy unit, a significant milestone in Indian oncology. At a time when chemotherapy was still in its experimental stages globally, Dr. Shanta introduced it at the Cancer Institute. She developed treatment protocols that were tailored to the Indian context, focusing on cost-effectiveness and patient accessibility. Her efforts in this area significantly improved survival rates, especially for cancers such as leukemia and lymphoma. Recognizing the challenges faced by children with cancer, she established specialized pediatric oncology services at the Cancer Institute and saved countless young lives which also brought hope to families battling the disease. She initiated several programs at the Cancer Institute aimed at providing psychological counseling, nutritional support, and palliative care to patients [4].

Research and academic contributions

Dr. Shanta’s academic contributions were as remarkable as her clinical work. She authored numerous research papers which were published in national and international journals, significantly contributing to the understanding of cancer epidemiology, treatment, and prevention in India [5]. She conducted extensive studies on the incidences, regional variations, impact of socioeconomic factors, and patterns of cancer in India, which provided critical data for public health policy and planning. Dr. Shanta was deeply involved in research on breast and cervical cancer, two of the most common cancers among Indian women [6]. Her work in this area led to the development of early detection programs and awareness campaigns that had a lasting impact on public health. She was also interested in clinical trials and evidence-based treatment protocols. She initiated and led several clinical trials at the Cancer Institute, focusing on the efficacy and safety of various cancer treatments. Her work contributed to the development of standardized treatment protocols that were widely adopted across India. She published more than 95 papers in national and international journals and contributed chapters in oncology books [7]. Hospital-Based Cancer Registry and the population-based Madras Metropolitan Tumor Registry were established under the leadership of Dr. Shanta, as a part of the National Cancer Registry Program of the Indian Council of Medical Research [4].

Holistic medicine and treatment

Her approach to patient care was holistic, addressing the physical, emotional, and psychological needs of the patients. Dr. Shanta believed in treating the patient, not just the disease. She believed in making cancer care accessible to all, regardless of socioeconomic status. She believed that quality cancer care should not be a privilege but a right for every individual. She was a driving force behind the establishment of regional cancer centers across India. She believed that decentralizing cancer care was essential to reach the rural population and improve access to cancer treatment in remote areas.

Leadership, legacy, awards, and recognition

Dr. Shanta was a key figure in the development of India’s National Cancer Control Program. She advised the Indian government on various aspects of cancer control, including policy formulation, program implementation, and resource allocation. Her input was instrumental in shaping the country’s cancer control strategy. She was a member of several expert committees and advisory boards. She served on several World Health Organization (WHO) expert committees on cancer contributing to the development of global cancer control strategies. She also collaborated with international cancer research organizations, bringing the latest advancements in cancer care to India.

Dr. Shanta’s contributions to medicine and public health were recognized with numerous awards and honors. She was awarded India’s highest civilian honors - Padma Shri in 1986, Ramon Magsaysay award for public service in 2002, Padma Bhushan in 2006, Avvaiyar Award by the Government of Tamil Nadu in 2013, and Padma Vibhushan in 2016 [7]. Despite these accolades, Dr. Shanta remained humble and focused on her mission to serve humanity. She trained and mentored countless oncologists, many of whom have gone on to become leaders in their fields. For all these efforts, Dr. V. Shanta will ever be remembered as the Mother of Oncology in India [8].

## Conclusions

The impact of Dr. Shanta’s work extends far beyond her lifetime even after her demise at the age of 93 on January 19, 2021. Adyar Cancer Institute continues her work in the field of oncology by serving cancer patients as pioneers in cancer care from prevention to palliation, offering ethical and state-of-the-art multi-modality treatment. Dr. V. Shanta’s life was a testament to the power of dedication. She was a compassionate healer and visionary who dedicated her life to fighting cancer and improving the lives of those affected by it. She was a pioneer who broke new ground in cancer treatment and care, and who touched countless lives. Her legacy lives on in the countless patients she helped and the institution she reformed. Dr. Shanta’s life and work serve as an inspiration to all, not just in the medical field but across society. Her commitment to service, her compassion for those suffering, and her relentless pursuit of excellence are values that resonate with those seeking to make a positive difference in the world.
